# Exploring the Characters of Non-Coding RNAs in Spermatogenesis and Male Infertility

**DOI:** 10.3390/ijms26031128

**Published:** 2025-01-28

**Authors:** Qiu Yan, Qi Wang

**Affiliations:** 1 College of Veterinary Medicine, Gansu Agriculture University, Lanzhou 730070, China; 18776410232@163.com; 2 Gansu Key Laboratory of Animal Generational Physiology and Reproductive Regulation, Lanzhou 730070, China

**Keywords:** non-coding RNAs, spermatogenesis, male infertility, Sertoli cells, Leydig cells

## Abstract

Infertility is a widespread clinical problem that affects human reproduction and species persistence worldwide. Around 40–70% of cases are due to male reproductive defects. Functional spermatogenesis (sperm production through several coordinated events) is at the heart of male fertility. Non-coding RNAs (ncRNAs) are the primary regulators of gene expression, controlling extensive critical cellular processes, for example proliferation, differentiation, apoptosis, and reproduction. Due to advancements in high-throughput sequencing tools, many studies have revealed that ncRNAs are widely expressed in germ cells, meiosis, spermatogenesis, sperm fertility, early post-fertilization development, and male infertility. The present review examines the biology and function of ncRNAs, including microRNAs, circular RNAs, and long ncRNAs, in spermatogenesis, their correlation with infertility, and their potential as biomarkers for sperm quality and fertility. The function of ncRNA in Sertoli cells (SCs) and Leydig cells (LCs) is also outlined throughout this study, because spermatogenesis requires testicular somatic cells to be involved in testicular development and male fertility. Meanwhile, the future development of ncRNAs for the clinical treatment of male infertility is also anticipated and discussed.

## 1. Introduction

In recent years, infertility has become a more and more widespread condition in industrialized countries, among which infertility in men has increased by about 0.291% annually [1]. On the one hand, infertility not only places a severe psychological burden on the patient but also affects the family happiness index. On the other hand, infertility and subfertility account for a large economic loss in the animal industry, particularly for rare and protected species. Infertility severely restricts the progress of breeding efforts [2]. Infertility results from impaired male and/or female gamete production or the inability of the gametes to reach each other or fuse. In this process, male factors contribute significantly, comprising 40.0% to 50.0% of infertility cases [3], and in a study of 4173 seeking infertility treatment, 63.0% had abnormal semen parameters. The causes of male subfertility or infertility are wide ranging and can be stratified as congenital, acquired, and idiopathic. Based on a detailed investigation report from one seminar, we subdivide these reasons in Table 1 [4].

The long-held belief in molecular biology was that messenger RNA (mRNA) provides the blueprint for translating proteins, while DNA is a template for mRNA transcription [5]. Eventually, a fresh understanding of this idea emerged with the fast-accelerating development of high-throughput techniques. Our knowledge of the coding and non-coding regions of the mammalian transcriptome has increased, along with the advancement of high transcriptome sequencing technology. Studies have discovered that although a significant portion of the human genome is translated into RNA, only approximately 1.9% encodes proteins [6,7]. While ncRNA cannot be directly translated into proteins, it can regulate protein synthesis, modify DNA, affect genes and epigenetics, and play an essential role in miscellaneous cellular events [5]. The elevated RNA expression levels in germ cells, sperm, and testicular somatic cells underscore the significance of non-coding RNAs (ncRNAs), including microRNAs (miRNAs), circular RNAs (circRNAs), and long ncRNAs (lncRNAs), in male infertility, particularly during spermatogenesis [8,9]. Spermatogenesis is an essential component of male reproductive physiology, and its dysfunction is the principal cause of male infertility. Spermatogenesis is a highly coordinated process governed by multilevel regulation of gene expression. To carry out the continuous production of spermatozoa from the spermatogonial stem cells (SSCs), it maintains an equilibrium between cell differentiation and proliferation through a full train of transformational processes, including mitosis, meiosis, and cell differentiation [10]. Spermatogenesis is inextricably linked to the Sertoli cells (SCs) and Leydig cells (LCs), the primary somatic cells of the testis, in such a multicellular process [11]. Yet, a great many latent ncRNAs have been in the news regarding spermatogenesis and male infertility; however, these ncRNAs have seldom or never been validated and functionally characterized. Unlike mRNA, ncRNAs are poorly understood in mammalian spermatogenesis, testicular development, male reproductive physiology, and infertility [12]. This article explores the characteristics of ncRNAs in spermatogenesis and the development of testicular somatic cells. Furthermore, the possible uses of ncRNAs for regulatory roles in diagnosing and treating male infertility are discussed.

**Table 1:** List of various reasons and associated risks for infertility or subfertility in men [4,13].

Congenital Factors	Acquired Factors	Idiopathic Risk Factors
Anorchia	Varicocele	Smoking
Congenital absence of vas deferens	Testicular trauma or torsion	Alcohol
Cryptorchidism	Systemic diseases	Recreational drugs
Y chromosome microdeletions	Germ cell tumors	Obesity
Chromosomal abnormalities genetic abnormalities	Acquired hypogonadotropic hypogonadism Postinflammatory conditions (epididymitis, mumps, orchitis)	Psychological stress Dietary factors
Klinefelter syndrome and its variants	Recurrent urogenital infections (prostatitis, prostatovesciculitis)	Advanced paternal age
Kallmann syndrome Mild androgen insensitivity syndrome	Urogenital tract obstruction Anti-sperm antibodies	Environmental exposure to toxins (organic contaminants, industrial and environmental chemicals, heavy metals, organic solvents, pesticides and endocrine disrupting chemicals)
Robertsonian translocation	Surgeries that can comprise vascularization of the testis Sexual dysfunction (erectile or ejaculatory dysfunction) Exogenous factors (chemotherapy, medications, radiation, heat) Systemic diseases (live cirrhosis, renal failure)	Occupational exposure to toxins

## 2. Spermatogenesis and Male Infertility

**Figure 1:**
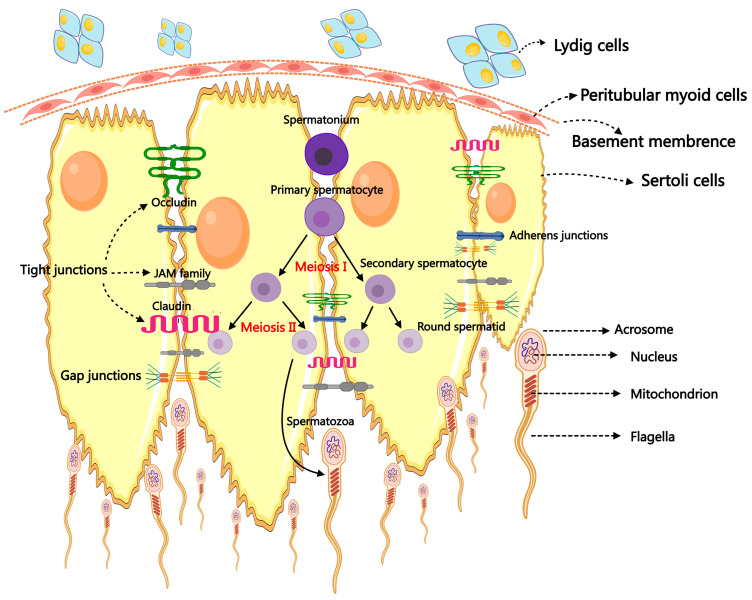
The primary mechanism of spermatogenesis and the morphology of spermatozoa.

Male reproductive success depends on a healthy testis, and spermatogenesis is the primary and crucial step in the physiological event of testicular reproduction [14]. The male fertility of mammals relies upon the ceaseless daily output of hundreds of millions of spermatozoa. The process, which begins in the seminiferous tubules (STs) and ends with the development of mature male gametes, is incredibly intricate and well-coordinated [15]. The additional subdivisions of spermatogenesis are spermatidogenesis, spermiogenesis, spermatocytogenesis, and spermiation. Spermatogenesis, which includes spermatogonial differentiation into spermatocytes, spermatocytes’ meiotic division that produces spermatids, the maturation of round spermatids, and the release of highly specialized mature spermatozoa into the ST lumen, was reviewed in references [16,17]. During spermatogenesis, the stem germ cell initially undergoes mitotic division, producing spermatogonia that participate in spermatogenesis. Then, the spermatogonia proliferate and produce preleptotene spermatocytes via several continuing mitotic divisions. The preleptotene spermatocytes cross the blood–testis barrier (BTB) and use meiotic prophase to produce round haploid spermatids [11]. Finally, spherical spermatids undergo differentiation into elongated spermatids and then into spermatozoa, which are discharged into the seminiferous tubule lumen during the process known as spermiation [11]. The differentiation of spermatogonia into spermatozoa involves a complex interplay of various cells, paracrine factors, hormones, genes, and epigenetic regulators, ultimately leading to the proliferation of SSCs and their maturation into specialized, terminally differentiated spermatozoa [18,19]. This process involves collaboration among testicular somatic cells, including SCs, LCs, and peritubular myoid cells (PMCs) [19,20]. Both germ cells and SCs undergo massive structural and morphological changes during spermatogenesis [21]. In particular, SCs undergo fierce morphological changes to accept a terminally differentiated state capable of supporting germ cell development [22] (Figure 1).

Spermatogenesis is a vital and complex physiological process for male reproduction. Spermatogenic failure (SPGF) is a clinical disease of fertility disorder. The leading causes of SPGF are the specific gene mutations from meiosis, mitosis, or spermiohistogenesis. SPGF can lead to oligozoospermia, azoospermia, asthenozoospermia, teratozoospermia, or a combination thereof [23]. This underscores the requirement for a complete evaluation of spermatogenesis processes, including protein-coding messenger RNAs and ncRNAs functions, to provide new insight into a cure for male infertility.

## 3. The Non-Coding RNA Family

It is well established that biological RNA includes coding RNAs and ncRNAs, of which ncRNAs that do not encode proteins account for the vast majority. The range of functions that ncRNAs can perform to regulate various aspects of life is extensive. It includes epigenetic control over gene expression [24], promoter-specific gene regulation, nuclear architecture maintenance [25], modification of DNA, etc., meaning ncRNAs play an essential role in regulating a variety of life activities [26].

**Figure 2:**
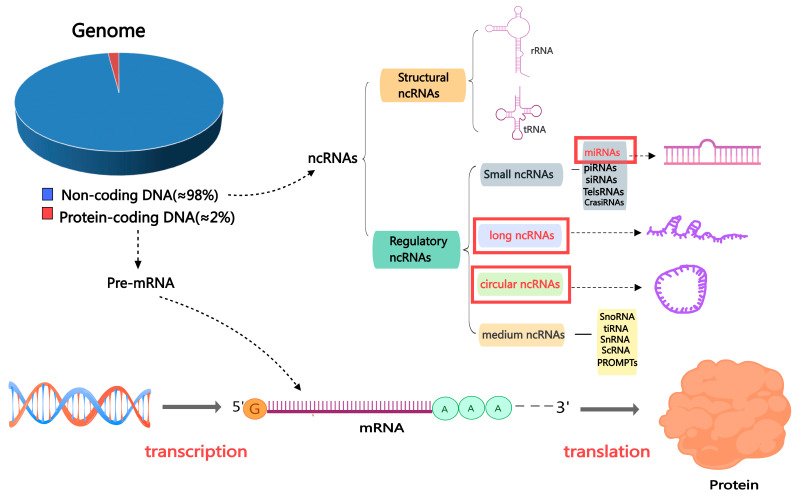
The classification of non-coding RNAs. Abbreviations: mRNA: messenger RNA; miRNA: microRNA; ncRNA: non-coding RNA; piRNA: Piwi-interacting RNA; siRNA: small interfering RNA; TelsRNA: telomere-specific small RNA; CrasiRNA: centromere repeat-associated small interacting RNA; snoRNA: small nucleolar RNA; scRNA: small cytoplasmic RNA; snRNA: small nuclear RNA; PROMPTs: promoter upstream transcripts; TiRNA is also known as tRNA halves; tRNA: transfer RNA.

ncRNAs are categorized into two classes based on their length. Those shorter than 200 nucleotides are called small or short non-coding RNAs (sncRNAs), while those greater than 200 are termed lncRNAs. Constitutive and regulatory sncRNAs can be distinguished among sncRNAs; regulatory sncRNAs include miRNAs and PIWI-interacting RNAs (piRNAs) [27]. In addition, circRNAs are a newly discovered category of ncRNAs, and their main feature is that the sequence is linked end to end to form a circular structure [28,29] (Figure 2). In this review, we concentrate on three primary and significant categories of ncRNAs that have garnered substantial interest: miRNAs, circRNAs, and lncRNAs. These three ncRNA types have been widely confirmed to regulate all transcriptional, post-transcriptional, or epigenetic levels as gene expression regulators. Most importantly, ncRNAs have regulatory roles in the reproductive process, such as in testicular development, sperm function, sperm maturation, and spermatogenesis [30,31]

## 4. The Function of ncRNAs in Spermatogenesis

The process of cell development, known as mammalian spermatogenesis, is a highly intricate physiological event that starts with the self-renewal and differentiation of stem cells [19]. The exact and space–time-specific regulation of gene expression at the transcriptional, post-transcriptional, and epigenetic levels is essential for the orderly progression of spermatogenesis.

During spermatogenesis, some stages occures many post-transcriptional regulatory events, the stages of primordial germ cell (PGC) formation and meiotic DNA recombination [32]. For example, in drosophila, nearly a quarter of the genome is transcribed in or near male-specific genes, and about 60% of the genes are expressed in the testis [33]. Mammalian testicular development and spermatogenesis are also regulated by many ncRNAs [31]. Thus, testicular tissue is an organ with strong transcriptional activity.

The intricate regulation and expression of genes in the testes primarily reflect the highly coordinated, delicate, and dynamic process of continual cell proliferation and differentiation in spermatogenesis. In addition, studies have shown that more than 1500 RNA-binding proteins (RBPs) and ncRNAs, including miRNAs and lncRNAs, are specifically highly expressed in the testis [34]. These RBPs assemble with ncRNAs to form RNA regulatory complexes, which play an indispensable role in spermatogenesis by regulating the fate of gene expression in male germ cells at multiple levels [9,35]. What is noteworthy is that recent basic and clinical evidence suggests that abnormal RNA regulation may be a new cause of male infertility [10].

### 4.1. MicroRNAs in Spermatogenesis

**Figure 3:**
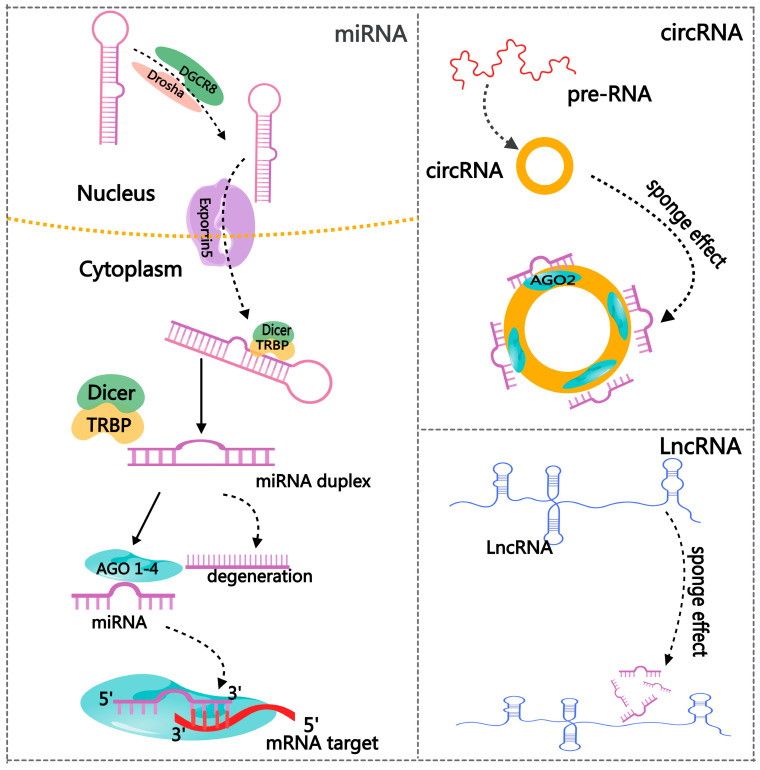
The regulatory network of non-coding RNAs.

The discovery of miRNA was a revolutionary breakthrough in the history of molecular biology. Lin-4 was the first miRNA and was described initially in *Caenorhabditis elegans* in 1993 [36,37]. miRNAs have a length of roughly 19–25 nt and are quite evolutionarily conserved. Furthermore, they belong to a class of endogenous single-stranded ncRNAs [38]. One of the most important and widely studied modes of action of miRNAs in organisms is repressing mRNA or hindering translation. However, miRNAs can interact directly and sequence-specifically with complementary target sites found in the 3′-untranslated region (3′-UTR) of mRNAs (Figure 3). Therefore, post-transcriptional regulation of gene expression occurs [38]. Moreover, miRNAs can silence cytoplasmic mRNAs via accelerating mRNA de-capping or promoting translation repression [39]. Over 60% of mRNAs possess miRNA target sites inside their 3′UTR regions. This suggests that the tight regulatory roles of miRNAs are extensive and are distributed in normal cellular homeostasis and various physiological events [40].

The destiny determinants of SSCs are crucial for preserving spermatogenesis, encompassing differentiation, self-renewal, and apoptosis. Some studies have found that miRNAs contribute to the adjustment of the status of SSCs. According to high-throughput sequencing, miR-21 is selectively expressed in SSC-enriched populations and is necessary to renew SSCs. Depressed miR-21 may enhance germ cell death while decreasing the potency of SSCs [41]. MiR-20 and miR-106a are predominantly expressed in SSCs in mice and are necessary for their renewal [42].

Moreover, miR-146 participates in modulating retinoic acid-induced spermatogonial differentiation [43]. The detrimental effects of miR-221/miR-222 promote differentiation and diminish stem cell potential [44]. Furthermore, the ablation of the mir-17-92 cluster in mice leads to reduced testicular size and a decreased sperm count in the epididymis [45]. Several investigations have demonstrated miRNAs’ crucial expression and functions in the control and differentiation of SSCs. The above data suggest that miRNAs can be expressed in different stages of cell differentiation, tissue development, and disease. As a result, miRNAs exhibiting distinct expression patterns may serve as diagnostic and therapeutic instruments associated with particular illnesses [46].

Furthermore, numerous animal studies have demonstrated that germ cell miRNAs regulate apoptosis, proliferation, and differentiation. Therefore, changes in miRNA expression patterns may hinder spermatogenesis by modulating cellular growth conditions [34,47]. Thus, miRNAs are post-transcriptional regulators and their functions involved with regulating all stages of spermatogenesis are significant.

### 4.2. CircRNAs in Spermatogenesis

As novel molecules of gene regulation, circRNAs have recently gained much attention. CircRNAs are more stably expressed than linear RNAs because of their covalently closed-loop structures. They are classified into three main categories: exon–intron circRNA, intron circRNA, and exon-derived circRNA [48]. Deep sequencing and other next-generation sequencing methods give us a fresh view of the diverse spectrum of circRNA expression levels, specificity, and variety in various tissues and developmental stages of eukaryotic organisms [49]. The primary function mechanism of cirRNAs is to act as miRNA sponges, regulating RBP activity and protein translation [50] (Figure 3). A recent study demonstrated that the circRNA testis-specific sex-determining region Y (Sry) functions as a miR-138 sponge and is crucial in sex determination [51].

Because of their incredible spatiotemporal specificity, circRNAs are essential for evaluating different mammalian tissues, organs, and developmental phases [52]. In the testes of neonatal (1-week-old) and adult (4-year-old) cattle, for instance, there is a difference in the expression of circRNAs such as piwi-like RNA-mediated gene silencing 1 (PIWIL1) and spermatogenesis-associated protein 6 (SPATA6) [28]. These expression patterns are critical for the development of spermatids and male fertility. [53,54]. Moreover, recent evidence suggests that the testes, second only to the brain, contain the most tissue-enriched circRNAs [28]. Testicular transcript sequencing research shows that human and mouse testes and seminal plasma have large circRNAs [55,56].

In recent times, male infertility has escalated to become a global concern in the reproductive domain. Further investigation has revealed the involvement of circRNAs in various pathways linked to spermatogenesis and male infertility, including asthenozoospermia, azoospermia, and oligospermia [57,58]. In these reproductive events, circRNAs can sponge miRNAs to remove the inhibitory role of miRNAs on their target gene expression and protein translation; also, circRNAs can act as protein decoys, and regulate biological pathways, or act as transcriptional regulators [58].

CircRNAs represent a novel category of molecular biomarkers for therapeutic and pharmacological applications in male infertility, particularly those circRNAs originating from genes that regulate spermatogenesis and sperm parameters [58]. A recent discovery identified 1017 host genes in the human testis that are intricately associated with spermatogenesis and have the potential to produce circRNAs [55]. CircRNAs exhibit superior stability and abundance compared to linear RNA, enabling substantial quantities of testis-derived circRNAs to be maintained as protein complexes in seminal plasma at ambient temperature. As a result, these testis-derived circRNAs may serve as non-invasive indicators for predicting male sperm quality and fertility and possess significant potential as liquid biopsy tools for various disorders [55]. Studies on circRNAs regulating spermatogenesis have mushroomed in recent years. Zhu et al. determined that circRNAs function in spermatogenic cell development by disrupting the mutual effects between SCs and the testicular immune microenvironment [59]. ssc_circ_0839 results from interactions between the genes and proteins necessary for male germ cell maturation during late spermatogenesis with the translation inhibitor PAIP2 [57]. Seminal plasma samples from idiopathic non-obstructive azoospermia (INOA) patients showed a lower expression level of hsa_circ_0049356 when compared to healthy controls. On the other hand, NOA patients showed an upregulation of hsa_circRNA_0023313, suggesting that circRNAs may be a biomarker for NOA and act as a regulator in spermatogenesis [60,61]. In the future, circRNA expression profiles could be a promising diagnostic method for many forms of male infertility.

### 4.3. LncRNAs in Spermatogenesis

LncRNAs, which have a minimum length of 200 nucleotides [61], serve many purposes in mammals [62]. LncRNAs suppress the expression of antisense mRNA and control the genes involved in chromatin remodeling. Furthermore, lncRNAs work as competing endogenous RNAs (ceRNAs) to suppress the activity of miRNAs and associate with DNA-binding proteins to obstruct their interaction with target genes [63] (Figure 3).

Although numerous putative lncRNAs have been found in male germ cell development, only a limited number have been functionally annotated and characterized; a concise review is provided in Table 2. For instance, the meiotic recombination hot-spot locus (MRH l) located in the nucleus is a monoexonic lncRNA [64] regulating spermatogenesis by two molecular mechanisms, one of which is the processing of MRH l by Drosha to form an RNA intermediate [65]. Furthermore, MRH l may obstruct the WNT signaling pathway by interacting with p68 [66]. Additionally, the Dmrt1-related gene (Dmr) is a functional lncRNA that is exclusive to the testis [67]; it upregulates Sohlh1 to enhance spermatogonial development [68,69]. Zhu et al. recently identified 1800 lncRNAs in human germ cells; 157 of them exhibited variable expression across several populations of testicular cells [70]. Following this, Rolland et al. discovered 113 known lncRNAs that are conserved across humans and rodents by comparing the lncRNAs in human germ cells with the RNA profiles of rat germ cells. Furthermore, specific, essential spermatogenic genes such FAM98B, KCNQ10T1, CENPB, RPGR, TPM2, and GNB5 are included in these prospective targets of lncRNAs [71]. Identifying these reproductive-related lncRNAs opens up new avenues for establishing regulatory connections with the pathways involved in spermatogenesis.

Furthermore, a study discovered that mice exhibiting particular lncRNA expression in a specific stage of spermatogenesis had a collection of lncRNAs with strong testicular expression patterns. Mice lacking the X-linked long non-coding RNA Tslrn1 (testis-specific long non-coding RNA1) showed a substantial decrease in the number of sperm; however, the underlying molecular pathways are as yet unknown [72]. Also, lncRNAs have different spatiotemporal expressions and an extensive repertoire of lncRNAs emerges during the maturation of mouse sperm [73]. The lncRNAs that Chen et al. determined to be expressed in mouse spermatogenic cells were more abundant and had the highest expression level in diplotene, M I spermatocytes [74]. In rats, chromosomes 5 and 9 co-transcribe the 1588 bp lncRNA HongrES2. HongrES2 is expressed in the cauda region of the epididymis. Overexpression of HongrES2 reduces sperm capacity, indicating that low endogenous lncRNA expression levels are a gatekeeper to the normal sperm maturation process in the epididymis and that low HongrES2 expression promotes sperm maturation [75]. For a long time, lncRNAs have been widely studied in mouse or rat sperm cells, but there is a lack of attention to large domestic animals. Wang et al. conducted an extensive examination of lncRNAs and mRNAs in high- and low-motility sperm of Holstein bulls, discovering that the lncRNA TCONS_000417333 targets the gene EFNA1, which is implicated in sperm motility and influences male reproductive physiology. This investigation represents a significant advancement in identifying bull sperm motility and fertility lncRNA markers, which have potential applications in animal husbandry [76].

Although public lncRNA annotation tools and advanced approaches have helped identify many viable lncRNAs, functional annotation of lncRNAs in spermatogenesis is still in its infancy but has enormous potential for the future. LncRNAs implicated in spermatogenesis and male infertility can be identified using publicly accessible lncRNA annotations; nevertheless, their precise localization and biological activity in the testes necessitate unique experimental data for validation [12].

**Table 2:** LncRNAs that have been shown to participate in spermatogenesis.

Name	Functions	Reactive Sites	Organism	Reference
NLC1-C	Inhibiting apoptosis promotes cell growth	Spermatogonia, spermatocytes	Human	[77]
Tug1 lncRNA	Regulating sperm numbers and morphology	Sperms	Human, Mouse	[78]
Mrhl	Cell adhesion, differentiation, signaling, and development	Spermatogonial cells	Mouse	[66]
Tsx	Regulating meiosis	Pachytene spermatocytes,	Mouse	[79]
Tesra	Regulating meiosis	LCs	Mouse	[80]
Drm	Regulating switching between mitosis and meiosis	SCs, germ cells	Mouse	[69]
Spga-lncRNAs	Maintaining stemness of spermatogonia	Spermatogonia, pachytene spermatocytes, round spermatids	Mouse	[81]
lncRNA-Tcam1	Immune response	SSCs	Mouse	[82]
LncRNA033862	Regulating SSC self-renewal	SSCs, spermatogonia	Mouse	[83]
AK015322	Maintaining SSC self-renewal capacity, promoting the proliferation of SSCs	Germ cells	Mouse	[84]
Gm2044	Regulating germ cell transition, regulating meiotic progression	Pachytene spermatocytes	Mouse	[85]
HongrES2	Promotes sperm maturation	Sperms in epididymis	Rat	[75]

Abbreviations: LCs: Leydig cells; SCs: Sertoli cells; SSC: spermatogonial stem cells; NLC1-C: Narcolepsy candidate-region 1 gene; Mrhl: Meiotic recombination hot spot locus; Tsx: Testis-specific X-linked; Drm: Dmrt1-related gene; lncRNATcam1: LncRNA—testicular cell adhesion molecule 1; Tug1-lncRNA: Taurine-upregulated gene 1 lncRNA.

## 5. ncRNAs in Testicular Somatic Cells

Spermatogenesis is the basis for establishing and maintaining male reproduction, and abnormal spermatogenesis can lead to male infertility. The testis’s microenvironment or niche comprises somatic cells, such as LCs, myoid cells, and SCs, which are crucial for controlling appropriate spermatogenesis [86]. In recent studies, ncRNAs have been shown to regulate the proliferation and adhesion [87], maturation and hormone responses [88], etc., in testicular somatic cells, meaning that different ncRNAs can be used as biomarkers for the diagnosis of abnormality in testicular somatic cells and male reproductive disorders. Therefore, it is essential to have a deeper comprehension of the basic molecular processes of ncRNAs that underlie the operation of testicular somatic cells.

### 5.1. ncRNAs in Sertoli Cells

Because the expression of their genes coordinates all the stages of germ cell differentiation, SCs are crucial cells in the testis and, in fact, among the most remarkable cells in the vertebrate body [89]. Spermatogenesis is renowned for being a dynamic, well-organized, and intricate process. During spermatogenesis, SCs function as “nurse cells”, providing the physical support and nourishment necessary to develop several germ cell types [90]. They are also in charge of establishing an environment that is functionally appropriate for the growth and development of germ cells [91]. SCs, for example, secrete the proteins collagen and laminin, which are critical components of the extracellular matrix, create specialized junctions, and have a well-organized cytoskeleton [91]. The functions of stem cells are essential for the morphofunctional structure of the epithelium of seminiferous tubules and the preservation of spermatogenesis, germ cell development, and viability [89].

Recent research indicates that the alteration of ncRNA expression in Sertoli cells may impact male fertility, and these investigations have sought to elucidate the role of Sertoli cells in spermiogenesis and male fertility [92].

### 5.2. miRNAs in Sertoli Cells

The delicate regulatory mechanisms involved in producing proteins in SCs are still unclear. Numerous recent investigations have concentrated on miRNAs, crucial regulators of gene expression via translational regulation. Numerous miRNAs in SCs have been shown to serve as essential regulators in the phases of barrier and seminiferous epithelium cycles. These miRNAs are influenced by hormones, specifically androgens (ARs) and follicle-stimulating hormone (FSH) [93]. With the deepening of research, there is increasing evidence that miRNAs affect spermatogenesis and male fertility processes by controlling the function of SCs. According to early research on miRNA binding sites and molecular pathways in SCs, miRNAs primarily control BTB protein regulatory mechanisms and SC proliferation, maturation, and apoptosis [88].

A type of endoribonuclease called DICER is essential to the miRNA pathway. Precursor molecules are changed into mature miRNAs by DICER. Poor spermatid attachment to SCs and uneven apical ectoplasmic specializations between elongating spermatids and SCs were seen in Dicer1 knockout male mice [94]. Severe failures were observed in all three phases of spermatogenesis when DICER1 was inactivated [95]. Phagocytosis and apoptosis are needed for maintaining tissue homeostasis. Numerous experiments have demonstrated that SCs’ phagocytosis, autophagy, and apoptosis play a crucial role in germ cell formation and differentiation, with over fifty percent of spermatogenic stem cells being eliminated and resolved by SCs [96]. Furthermore, spermatogenesis collapsed, the multilayer architecture of the seminiferous epithelium was reduced, the incidence of apoptosis increased, and the number of STs decreased when DICER1 was conditionally knocked out in mouse SCs [97]. Furthermore, downregulation of genes, including Wilms tumor 1 protein (WT1), mannosidase alpha class 2A member 2 (MAN2a2), a ligand for the KIT tyrosine kinase receptor (KitL), and glial cell-derived neurotrophic factor (GDNF) was also a consequence of these DICER knockouts. Each of these genes has significant spermatogenesis-related characteristics [98]. In addition to this, DICER1-deficient testes exhibit an over-expression of the SOD 1 protein and a consequent increase in oxidative damage, resulting in aggressive apoptosis and testicular degeneration [99]. In particular, miR-125a-3p, miR-872, and miR-24 may target SOD-1 to cause cell apoptosis [99]. Dock180 is a component of LC3-dependent phagocytic complexes and interacts with autophagy-related proteins. By repressing the expression of Dock180, Atg12, LC3, Atg12, Beclin 1, and Rubicon, miR-471-5p damages male fertility by causing apoptosis in germ cells [100]. Mitogen-activated protein kinase 11 (MAPK11) activation in SCs may stimulate the expression of tumor necrosis factor α (TNF-α), which binds to and activates TNF receptor 1 (TNFR1), ultimately leading to the death of germ cells. According to a study, the 3′ UTR of MAPK11 was predicted to be the binding site for miR-758 and miR-98-5p [101].

Not only do SCs function as feeder cells, offering nourishment and support to diverse germ cells, but they are also critical components of the BTB, a crucial ultrastructure for male fertility [102]. BTB, which is made up of gap junctions (GJs), adherent junctions (AJs), tight junctions (TJs), and desmosome-like junctions, is one of the most impenetrable blood–tissue barriers in the living body [103]. BTB is essential for spermatogenesis and male fertility because it is a physical barrier to spermatogenesis activities in an immune-privileged environment [104]. Numerous studies have demonstrated the critical role that the miR-17-92 cluster plays in mouse spermatogenesis [45]. One miR-17-92 cluster member, miR-20a, targets Limk1 to control the dynamics of AJs in SCs and germ cells and impact BTB functions via the RhoB/ROCK/LIMK1 pathway [105].

Since defects in SC maturation and proliferation regulation can cause particular testicular dysfunctions, the investigation of miRNAs may be crucial to better understand the molecular regulatory circuits underlying these mechanisms. Even though the information currently available points to a few potential molecular targets for SC miRNAs, a thorough understanding of SCs’ miRNA pathways is still necessary to treat infertile patients with a variety of etiologies, such as mixed atrophy, hypogonadism, and Sertoli cell-only syndrome (SCOS) [88].

### 5.3. circRNAs in Sertoli Cells

In eukaryotes, circRNAs are endogenous macromolecules that are covalently closed. Certain cis- and trans-acting factors regulate the synthesis of circRNAs following tissue- and cell-specific expression patterns. CircRNAs work as miRNAs or protein sponges to carry out their essential biological roles by regulating or translating protein functions. Multiple studies have demonstrated the connection between circRNAs and diseases, including cancer, neurological conditions, cardiovascular diseases, and diabetes mellitus. However, more research on the particular regulatory systems is still needed [106]. Zhu et al. investigated the expression patterns of circRNA in the testicular tissues of individuals with SCOS and their potential activities. High-throughput microarray analysis of circRNAs revealed that 399 circRNAs are upregulated and 1195 are downregulated in SCOS relative to obstructive azoospermia. These differentially expressed circRNAs, which target biological processes linked to immune cell formation, intercellular communication, and the cell cycle, are substantially expressed in SCs [59]. By binding to the 3′ UTR of RIG-I and TLR3, respectively, miR-136 and miR-26a suppressed the expression of retinoic acid-inducible gene-I (RIG-I) and Toll-like receptor 3 (TLR3) in SCs and LCs, respectively; circRNA-9119 acts as a miRNA sponge of miR-136 and miR-26a to mediate inflammatory reactions and influence the immune microenvironment of testis [107]. In addition, circELAVL2 promotes cell proliferation and suppresses cell apoptosis in mouse TM4 SCs by binding to and inhibiting miR-382-3p, implying that circELAVL2 contributes to the maintenance of a spermatogenic environment in mice [108]. Overall, abnormal expression of circRNAs may regulate the functions of SCs and the spermatogenic microenvironment. However, the current research on cyclic RNA and mammalian reproductive physiology is relatively lacking and investigating this will require significant future research.

### 5.4. LncRNAs in Sertoli Cells

Sertoli cells are essential for spermatogenesis in the STs by forming BTB and creating a unique microenvironment for spermatogenesis. Meanwhile, the character of lncRNAs in SCs has infrequently been reviewed. A study found that the lncRNA Tug1 is implicated in BTB disruption, as its depletion significantly impairs the TJs of SCs. This finding provided a new insight to understand the role of lncRNAs in male infertility [109]. Similarly, a study in mouse testes showed that knocking out lncRNA5251 enhanced the expression of genes for cell junctions such as JAM1, VCAM1, CX37, OCLN, and CADM2, implying that lncRNA5251 is involved in spermatogenesis by modulating cell junctions [110]. Spermatogenesis and the development of male reproduction are directly impacted by the capacity of SCs to proliferate and differentiate. It is worth noting that WNT proteins play a role in controlling SC proliferation and differentiation and that variations in Connexin43 expression accompany variations in WNT expression. Connexin43 is a gap junction molecule essential for germ cell development that is associated with infertility and oligospermia [111]. A novel study performed high-throughput sequencing of testicular tissue from Dazu black goats at neonatal, early puberty, and sexual maturity, respectively, and found that lncWNT3-IT, as a target of WNT3, is expressed in the cytoplasm of SCs.

Furthermore, lncWNT3-IT can positively modulate WNT3 expression, affecting SC proliferation in the G0/G1 phase [112]. Important receptors for SCs are androgen receptors. The testis tissues of Dazu black goats at various developmental stages were subjected to high-throughput sequencing and bioinformatics analysis, which demonstrated that lncNONO-AS could regulate the production of AR by regulating the expression of NONO [113]. After attaining reproductive maturity, aging is a general, slow, and progressive change in the organism that occurs during almost all physiological processes. Aging regulation has been linked to lncRNAs. A recent study suggested that the molecular physiology of adult testes’ main spermatocytes, LCs, and SCs depends on the expression of the long non-coding RNA LINC-RSAS. Reduced LINC-RSAS correlates with diminished testicular function in aging rats [114]. Male germ cells express the Catsper1 gene necessary for sperm motility and fertilization. The polyadenylated long non-coding RNA (lncRNA) known as Catsper1au or 1402BP, is expressed in the nuclei of germ cells, LCs, and SCs, indicating that it may have an impact on male fertility and spermatogenesis. However, the specific mechanism is unknown and this needs more research [115]. Although their specific targets are unknown, many lncRNAs have been found in SCs [116].

Many studies have concluded that ncRNAs have a role in controlling SC function. Among these, ncRNAs in SCs are essential for immunological defense, cell growth, cell proliferation, and apoptosis etc., [117,118]. These ncRNAs are necessary for the blood–testis barrier, which maintains the testicular microenvironment for spermatogenesis [109,119]. However, numerous ncRNAs have been identified in SCs, and the research on reproductive function merely scratches the surface.

### 5.5. ncRNAs in Leydig Cells

Numerous studies have demonstrated that LCs are vital to managing SSC niches and play a crucial role in fetal testicular morphogenesis and stimulating spermatogenesis, influencing male fertility [120]. Through secreting ARs, other hormones, cytokines, growth factors, transcription factors, and receptors connected to LCs, the LCs initiate spermatogenesis [86]. Lack of ARs, such as testosterone (T), affects general health in males [121]. Furthermore, LCs play a significant role in sustaining secondary sexual traits and other elements of spermatogenesis [122]. Numerous ncRNAs are expressed in LCs, as assessed by contemporary investigations; in the following, we provide an overview of the research on the role of ncRNAs in LCs.

### 5.6. miRNAs in Leydig Cells

Leydig cells are a unique class of cells that, once created, seldom proliferate and rarely die; however, as they age, their ability to produce steroids gradually decreases. Male adult LCs are the primary source of testosterone; the hypothalamus–pituitary complex closely constrains their steroidogenic function–gonad axis and declines with age [123,124]. Primary fibroblast growth factor (bFGF) can stimulate the synthesis of AR [125] and encourage the commitment of stem LCs towards differentiation in LCs that produces testosterone [126]. There are at least five miRNAs regulated by bFGF that are involved in the regulation of AR production in LCs; they are miR-142-3p, miR-451, miR-29a, miR-29c and miR-335 [126]. The results reported here imply that variations in the expression of bFGF-mediated miRNAs may regulate the generation of ARs. These variations may also impact the signaling pathways involved in T biosynthesis and the expression of the steroidogenic gene. High-density lipoprotein (HDL) receptor class B type I (SR-BI) is required for the specific uptake of HDL cholesteryl esters in steroidogenic cells [127]. SR-BI expression and the selective absorption of HDL cholesterol esters were reduced following transfection of pre-miRNA-125a and pre-miRNA-455 in LCs, indicating a potential function for miRNA-125a and miRNA-455 in steroidogenesis in LCs [128]. Furthermore, the data suggest that the absence of miR-140-3p and miR-140-5p increases the number of LCs in mice, indicating a potential connection between these genes and the development of the mouse gonad and testicular differentiation [107]. Furthermore, melatonin inhibits T synthesis by targeting miR-7481-3p/CXCL14 and inhibiting the PI3K/AKT pathway. miR-29a blocks the function of the AR receptor and its target genes via the IGF-1 and p53 pathways [129], and miR-150 negatively regulates the expression of STAR and steroidogenesis of LCs [130]. In comparison, melatonin inhibits T synthesis by targeting miR-7481-3p/CXCL14 and inhibiting the PI3K/AKT pathway [131].

The results of this study might offer some fresh perspectives on miRNAs as putative therapeutic targets for the steroidogenesis regulating mechanisms in LCs and disorders linked to LC dysfunction. Even though miRNAs are essential for spermatogenesis, little is known about how miRNAs regulate spermatogenesis in LCs, and further research on this topic will require a significant amount of work.

### 5.7. circRNAs in Leydig Cells

A novel type of non-coding RNAs, circular RNAs are strongly expressed in eukaryotes and frequently exhibit cell- or tissue-specific expression patterns. Furthermore, they are more stable and conservative due to their circular construction [132]. A testis-enriched circRNA known as circ-Bbs9 was found in one study. It is strongly expressed in mouse LCs and regulates Ccnd2 levels, which are related to the cell cycle and essential to LC proliferation [133]. According to a different study, circRNA-9119 functions as a competing endogenous RNA to regulate the production of inflammatory cytokines in the testes and protects RIG-I and TLR3 mRNAs from being inhibited by miR-26a/miR-136, which in turn prevents LCs and SCs from growing [107]. On the other hand, little has been discovered about the regulating mechanism of circRNA in LCs in other animals.

### 5.8. LncRNAs in Leydig Cells

It has been observed that non-coding RNAs play significant roles in regulating genes (transcriptional, post-transcriptional, or post-translational) and various biological processes, such as the immune response, cancer, and cell proliferation [134]. Environmental pollutants significantly contribute to testicular injury and have been demonstrated to modify lncRNA expression patterns in the testis [135,136]. A study has confirmed that exposure to DEHP/MEHP leads to an accumulation of ROS and accelerated cellular aging of LCs, activating the antioxidant system. Moreover, the lncRNA-miRNA-mRNA competing ceRNA networks may significantly influence the regulation of FOXO signaling and longevity pathways in response to elevated ROS levels and the increased aging of LCs [137]. Besides regulating cell senescence, lncRNAs have also been shown to influence the apoptosis of LCs. A study indicated that the lncRNA MIR22HG facilitates LC apoptosis by functioning as a competitive endogenous RNA for microRNA-125a-5p [138], whereas the lncRNA FENDRR enhances LC apoptosis by accelerating the degradation of Nrf2 [139].

Leydig cells are essential for the precise regulation of spermatogenesis. Reproductive hormones, including testosterone, are synthesized by Leydig cells and are necessary for meiosis and spermatogenesis. A study on sheep reproductive physiology identified 33,883 lncRNAs from sheep testes. Among them, the lncRNA TCONS_00863147 could interact with PRKCD in a transactivation mechanism and affect spermatogenesis [116]. The steroidogenesis-activating lncRNA in testis (Start) was primarily localized in the cytoplasm of Leydig cells, serving as a regulator of steroidogenesis [140]. The lncRNA CIRBIL was 862 nucleotides in length and was predominantly localized in the cytoplasm of LCs, with a minor fraction within the STs. CIRBIL is a regulator of steroid hormone synthesis, for the reducing of testosterone levels in serum and expression of testosterone biosynthesis genes such as STAR and 3β-HSD in the CIRBIL-KO mice [141].

An increasing number of ncRNAs have been found in LCs due to the advancement of large-scale genomic technology and bioinformatics analysis. A growing body of studies has demonstrated the role of ncRNAs in steroidogenesis, testosterone synthesis, and LC development [129,141,142]. Several novel lncRNAs have recently been found, including Tesra, NLC1-C, Mrhl, HongrES2, and Tsx. These findings provide more insight into the regulation of gene expression, particularly concerning the regulatory mechanism of ncRNAs [12]. However, it must be acknowledged that the roles and mechanisms of ncRNAs in LCs concerning proper spermatogenesis and male infertility remain mostly unidentified. A comprehensive understanding of the function of the ncRNAs produced by LCs in regulating spermatogenesis and male reproduction is essential for identifying novel targets for male infertility treatment and developing innovative male contraceptive techniques.

## 6. Other Important ncRNAs in Spermatogenesis and Male Infertility

In addition to miRNAs, circRNAs, and lncRNAs, the known ncRNAs, PIWI-interacting RNAs (piRNAs), endogenous siRNAs, tRNA-derived small RNAs (tsRNAs), and rRNA-derived small RNAs (rsRNAs) have also been shown to be involved in spermatogenesis and male reproduction [143]. Among them, the study of piRNAs and male reproduction in particular has attracted much attention. Here we will briefly summarize the study.

PiRNAs are a class of small RNAs that interact with the PIWI clade of Argonaute proteins, directing PIWI proteins to silence transposons and regulate gene expression [144]. The Piwi clade consists of the HILI, HIWI1, HIWI2, and HIWI3 proteins in humans, Aubergine (Aub) and Ago3 in flies, and MILI, MIWI, and MIWI2 in mice. In mammals, piRNAs are mainly classified into two types based on their spike in expression at specific stages of spermatogenesis, namely pre-pachytene piRNAs expressed in spermatogonia and pachytene piRNAs expressed in pachytene spermatocytes [145].

Specific piRNA clusters are required for normal sperm function, and deletion of piRNA clusters on chromosome 18 results in acrosome dysgenesis, severe sperm head dysmorphology, and failure of fertilization due to impaired motility [146]. More importantly, the deletion of the piRNA cluster on chromosome 6 leads to defects in the acrosome response and sperm motility [147]. Intriguingly, many mutations in piRNA pathway factors lead to meiotic arrest during spermatogenesis. The significance of the piRNA pathway in male fertility has also been corroborated by the fact that the majority of piRNAs in factor-knockout animals exhibit arrested meiosis in spermatogenesis and only a few exhibit post-meiosis male germ cell arrest [148]. Knockouts of the PIWIL 1, PIWIL 4, and PIWIL 2 genes in mice have all resulted in complete spermatogenesis arrest at specific steps of differentiation. The number of germ cells was reduced in PIWIL l and 4 knockout mice. [149]. Mice deficient in PIWIL 2 had complete cessation of spermatogenesis [150]. Deletion of PIWIL 1 results in a later phenotype, with spermatozoa arrested in the early stages of spermatogenesis, demonstrating the essential role of PiWIL1 in regulating haploid differentiation and the morphological transformation of spermatophores into spermatozoa [151]. It has previously been reported that the MIWI/piRNA machinery is responsible for mRNA elimination during late spermatogenesis in preparation for spermatozoa production and that the MIWI/piRNA machinery is responsible for the activation of translation of a subset of spermatogenic mRNAs in coordination with morphological transformation into spermatozoa [152].

These findings not only reveal a critical role of the piRNAs system in translational activation, which is functionally required for spermatogenesis, but also suggest that piRNAs also hold great therapeutic potential for the treatment of male infertility.

## 7. Discussion and Conclusions

A disease of the reproductive system, infertility affects people worldwide and is a public health concern. A highly controlled biological process essential to male reproduction’s physiology is spermatogenesis. Most of the major causes of male infertility are due to the dysfunction of the spermatogenesis process, resulting in disturbances in sperm quality, such as motility and morphology, and a reduction in the amount of sperm in the semen. Unraveling the regulatory mechanisms of spermatogenesis is essential for the design of effective treatments for male infertility and male reproductive disorders.

The relationship between uncoded ncRNAs and spermatogenesis, fertility, and infertility has long been explored. Because ncRNAs are abundant and stable, there is a good chance that they can be used as trustworthy fertility biomarkers. Numerous studies have found numerous differentially expressed ncRNAs that link with infertility and have demonstrated dramatically changed expressions of ncRNAs in the testicular tissue, spermatozoa, and seminal plasma of infertile men. For instance, miRNAs have distinct functions in sperm, epididymis, seminal plasma, microvesicles, and other testicular tissues [153]. Any changes in the miRNA level may serve as biomarkers to assess the health of the spermatogenesis processes, which may lead to various forms of infertility.

**Figure 4:**
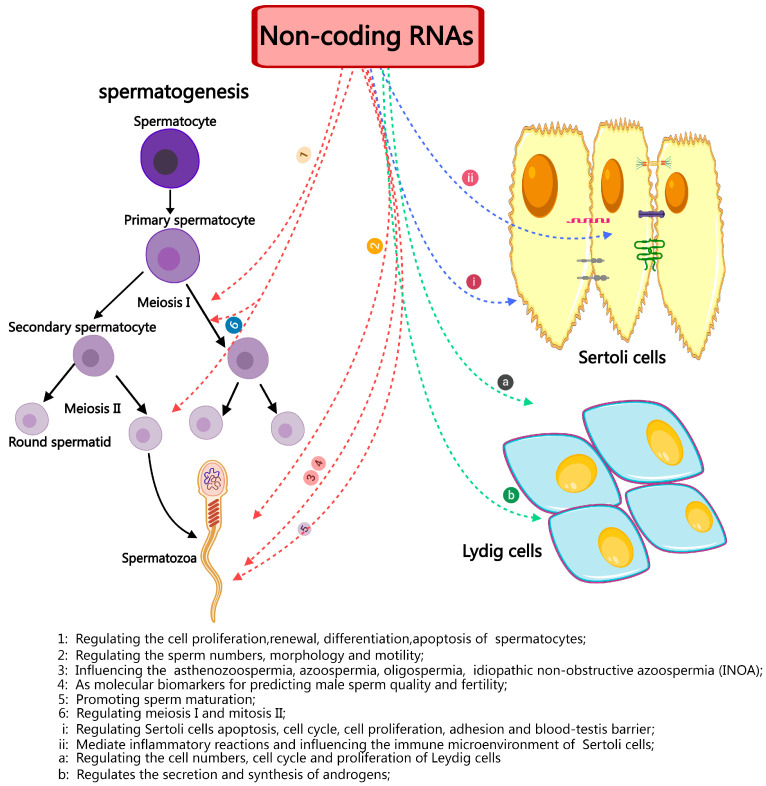
A brief summary of the role and importance of non-coding in spermatogenesis and male infertility.

Furthermore, a comprehensive investigation of ncRNAs, particularly miRNAs, has elucidated their fundamental significance in the development of germ cells and their involvement in critical processes such as sperm maturation, fertilization, and post-fertilization development (Figure 4). MiRNAs like hsa-miR-9-3p, hsa-miR-30b-5p, and hsa-miR-122-5p have the potential to serve as indicators of the fertility and quality of sperm. Similar to miRNAs, ncRNAs and circRNAs have only been the subject of a few studies but they have enormous potential for discovering new indicators of sperm quality and fertility. Therefore, identifying and characterizing the particular ncRNA signatures linked to infertility offers essential insights into the processes underlying spermatogenesis and could lead to discovering non-invasive diagnostic targets and targeted therapy agents for male infertility. Sperm gene expression and its function in numerous processes, including lumen formation during development, spermatocyte advancement through meiosis, spermatid maturation, and motility, are likely regulated by ncRNAs. Therefore, it is imperative to thoroughly investigate the regulation of spermatogenesis by ncRNAs from many angles and viewpoints. This includes identifying novel ncRNAs and assessing their unique methods of action.

Because of the recent review in ncRNA biology, we believe that the current understanding of ncRNA regulation in spermatogenesis and male infertility is incomplete, despite the numerous investigations that have recently introduced ncRNAs and confirmed their functions for regulating spermatogenesis. This is especially true for lncRNAs and circRNAs. The functional annotation of non-coding RNAs in spermatogenesis is still lacking, despite the production of diverse transcriptome data that have improved expression evidence for ncRNA prediction and annotation. Hence, higher-throughput data, more refined bioinformatics pipelines, and more comprehensive public databases are needed.

ncRNAs possess significant therapeutic potential for addressing male infertility as well. Currently, there is no research on the clinical therapy of infertility via ncRNAs. However, ncRNAs have demonstrated potential in treating various diseases, which we can discuss. Several miRNAs, such as miR-34c, miR-122, and miR-10b, have progressed to clinical trials for therapeutic use in diabetes and cancer [143,154]. These examples give us hope that there are areas where similar applications of ncRNAs to male infertility can be attempted in the future. The current investigation must concentrate on elucidating the complex role of ncRNAs in spermatogenesis and fertility to leverage their potential in comprehensively understanding and unraveling the regulatory mechanisms of spermatogenesis. We must acknowledge that we have only scratched the surface of the experience and uses of ncRNAs in spermatogenesis and infertility, with much remaining obscured beneath the surface.

## Data Availability

The datasets used and/or analyzed during the current study are available from the corresponding author on reasonable request.

## Abbreviations

ncRNA	non-coding RNA
lncRNA	long non-coding RNA
miRNA	microRNA
mRNA	messenger RNA
piRNA	PIWI-interacting RNA
tRNA	transfer RNA
rRNA	ribosomal RNA
SSCs	spermatogonial stem cells
LCs	Leydig cells
SCs	Sertoli cells
STs	seminiferous tubules
BTB	blood–testis barrier
PMCs	peritubular myoid cells
SPGF	spermatogenic failure
PGCs	primordial germ cells
RBPs	RNA-binding proteins
sncRNAs	small or short non-coding RNAs
